# Molecular evidence of a dominant multidrug resistant *Acinetobacter baumannii* lineage in a tertiary hospital in northern Peru

**DOI:** 10.1016/j.nmni.2026.101864

**Published:** 2026-10-10

**Authors:** Kevin G. Quispe Choque, Nataly Ruiz Quiñones, Milena Binatti Ferreira, Ana L. Perini-Leme-Giordano, Angélica Zaninelli Schreiber, Fabiana Fantinatti Garboggini, Fanny L. Casado, Rene Flores Clavo

**Affiliations:** a Bioengineering Section, Department of Engineering, Pontificia Universidad Católica del Perú, San Miguel, Lima, Peru; b Instituto de Ciencias Ómicas y Biotecnología Aplicada, Pontificia Universidad Católica del Perú, San Miguel, Lima, Peru; c Department of Biotechnology, Center for Research and Innovation in Multidisciplinary Active Sciences (CIICAM), Lambayeque, Peru; d Division of Microbial Resources of Chemical, Biological and Agricultural Pluridisciplinary Research Center (CPQBA), University of Campinas, Sao Paulo, Brazil; e School of Medical Sciences, University of Campinas, Campinas, Sao Paulo, Brazil

**Keywords:** *Acinetobacter baumannii*, Multidrug resistance, Clonal analysis, Molecular epidemiology, MALDI-TOF MS, Repetitive-element PCR, Intensive care unit, Peru

## Abstract

**Background:**

*Acinetobacter baumannii* is a major nosocomial pathogen because of its genomic plasticity and capacity to acquire multidrug resistance (MDR), particularly resistance to carbapenems. The World Health Organization classifies carbapenem-resistant *A. baumannii* as a critical-priority pathogen. Molecular epidemiological data from Latin America remain limited, especially for northern Peru.

**Methods:**

Drug-resistant *A. baumannii* isolates collected between 2015 and 2021 at a tertiary referral hospital in northern Peru were characterized by antimicrobial susceptibility testing, BOX-PCR, ERIC-PCR, REP-PCR, and matrix-assisted laser desorption/ionization time-of-flight mass spectrometry (MALDI-TOF MS).

**Results:**

All seven isolates met the criteria for MDR, and Ac-4268 showed an extensively drug-resistant phenotype. Repetitive-element PCR identified a group of six isolates with similar banding profiles and a divergent isolate, Ac-4268. Several isolates in the six-isolate group retained susceptibility to one or more aminoglycosides, whereas Ac-4268 was resistant to all antimicrobials tested. The combined molecular and phenotypic findings are consistent with the possible persistence of a dominant related lineage, but they do not establish transmission without whole-genome sequencing and epidemiological linkage data.

**Conclusion:**

This study provides additional molecular epidemiological evidence on MDR *A. baumannii* in northern Peru. The findings suggest that a dominant related lineage may have persisted in the hospital alongside a genetically divergent isolate with an extensively drug-resistant phenotype. Confirmation of clonal transmission requires higher-resolution genomic and epidemiological analyses.

## 1 Introduction

*Acinetobacter baumannii* is an aerobic, Gram-negative, non-motile coccobacillus of the class Gammaproteobacteria. It is a strict aerobe and is catalase-positive and oxidase-negative [1]. Members of the genus Acinetobacter occur in diverse environments, but *A. baumannii* is an opportunistic pathogen primarily associated with healthcare-associated infections. These infections disproportionately affect immunocompromised, older, and critically ill patients, particularly those with prolonged intensive care unit (ICU) stays or extensive antimicrobial exposure [2], [3]. Duan et al. reported high resistance rates to aminoglycosides, carbapenems, and cephalosporins among *A. baumannii* ST2 isolates [4]. *A. baumannii* causes pneumonia, bloodstream infection, urinary tract infection, meningitis, endocarditis, and skin, soft-tissue, and wound infections [5]. Its ability to colonize patients and healthcare workers and to survive on dry surfaces and medical equipment facilitates persistence in hospitals [6].

*Acinetobacter baumannii* shows intrinsic resistance to several antimicrobial agents and can acquire additional resistance determinants, which complicates treatment and contributes to hospital outbreaks [7], [8]. The species belongs to the ESKAPE group of highly drug-resistant nosocomial pathogens [9]. Its principal resistance mechanisms include reduced permeability, active efflux, modification of antimicrobial targets, and enzymatic inactivation. Virulence-associated traits may also contribute to persistence and reduced antimicrobial susceptibility in healthcare settings [10].

Nearly 60% of *A. baumannii* isolates recovered from hospitalized patients have been reported as multidrug-resistant [11]. Beta-lactamase production is a particularly important mechanism. A meta-analysis of New Delhi metallo-beta-lactamase-producing *A. baumannii* reported that 64.02% of isolates with this determinant originated in Asia, 28.05% in Africa, and 5.56% in Europe [12]. In South America, hospital-associated carbapenem-resistant *A. baumannii* populations include lineages related to ST15 and ST79 [13].

Resistance to colistin, a last-line polymyxin used against MDR nosocomial infections, is also a major concern. Quorum-sensing inhibition and quorum quenching are being investigated as potential strategies against colistin-resistant *A. baumannii* [14]. A systematic review estimated colistin-resistance prevalence at approximately 17% in Southeast Asia and the Eastern Mediterranean region, compared with 3% in the Americas [15]. ICU outbreaks may involve the expansion of highly resistant, genetically related *A. baumannii* isolates, making strain-level surveillance important for infection control [16].

In Peru, hospital isolates of *A. baumannii*, particularly those recovered from ICUs in Lima, show resistance to multiple antimicrobial classes [17]. Carbapenemase production is a frequently reported mechanism [18]. and several *A. baumannii* lineages circulate in the country [19]. Studies from Lambayeque and Trujillo reported *A. baumannii* in 27% and 17%, respectively, of hospital-derived bacterial isolate collections [20], [21].

Matrix-assisted laser desorption/ionization time-of-flight mass spectrometry (MALDI-TOF MS) provides rapid and reliable bacterial identification [22], [23]. Repetitive-element PCR methods can complement species identification by comparing genomic fingerprint patterns. ERIC-PCR has separated meropenem-resistant *A. baumannii* isolates into distinct clusters [24]. and BOX-PCR, REP-PCR, and ERIC-PCR have been used to assess genetic relatedness among hospital isolates [25].

The diversity and mobility of resistance determinants support the need to combine phenotypic susceptibility testing with molecular surveillance [26]. This exploratory study characterized antimicrobial resistance and genetic relatedness among *A. baumannii* isolates from a tertiary hospital in northern Peru by integrating repetitive-element PCR and MALDI-TOF MS profiling (Fig. 1).

**Fig. 1:**
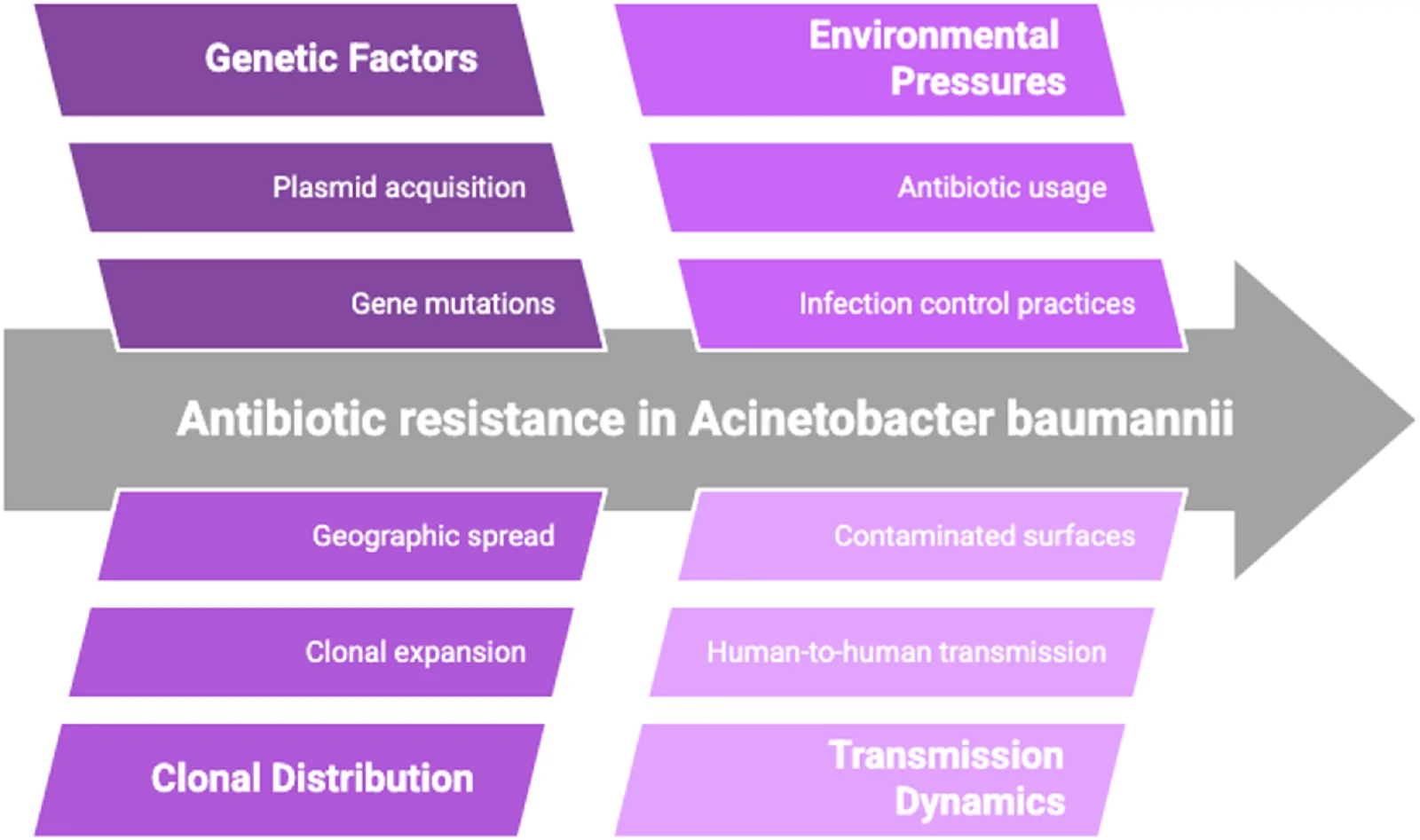
Schematic overview of the principal factors associated with antimicrobial resistance in *Acinetobacter baumannii*, including genetic determinants, environmental pressures, lineage distribution, and potential transmission pathways.

## 2 Materials and methods

### *2.1* Study design and isolate selection

Twenty-four MDR *A. baumannii* isolates were initially available from the study period. Preliminary ERIC-PCR and BOX-PCR fingerprinting showed that more than 70% grouped within one subclade with highly similar banding patterns. To reduce redundant characterization while retaining the diversity observed in the initial collection, seven isolates were purposively selected for full antimicrobial susceptibility testing, BOX-PCR, ERIC-PCR, REP-PCR, and MALDI-TOF MS analysis. The selected subset included representative isolates from the predominant subclade and isolates with divergent preliminary profiles (Fig. 2). Because selection was non-random and only seven isolates underwent complete characterization, the subset may not represent the distribution of lineages in the original collection.

**Fig. 2:**
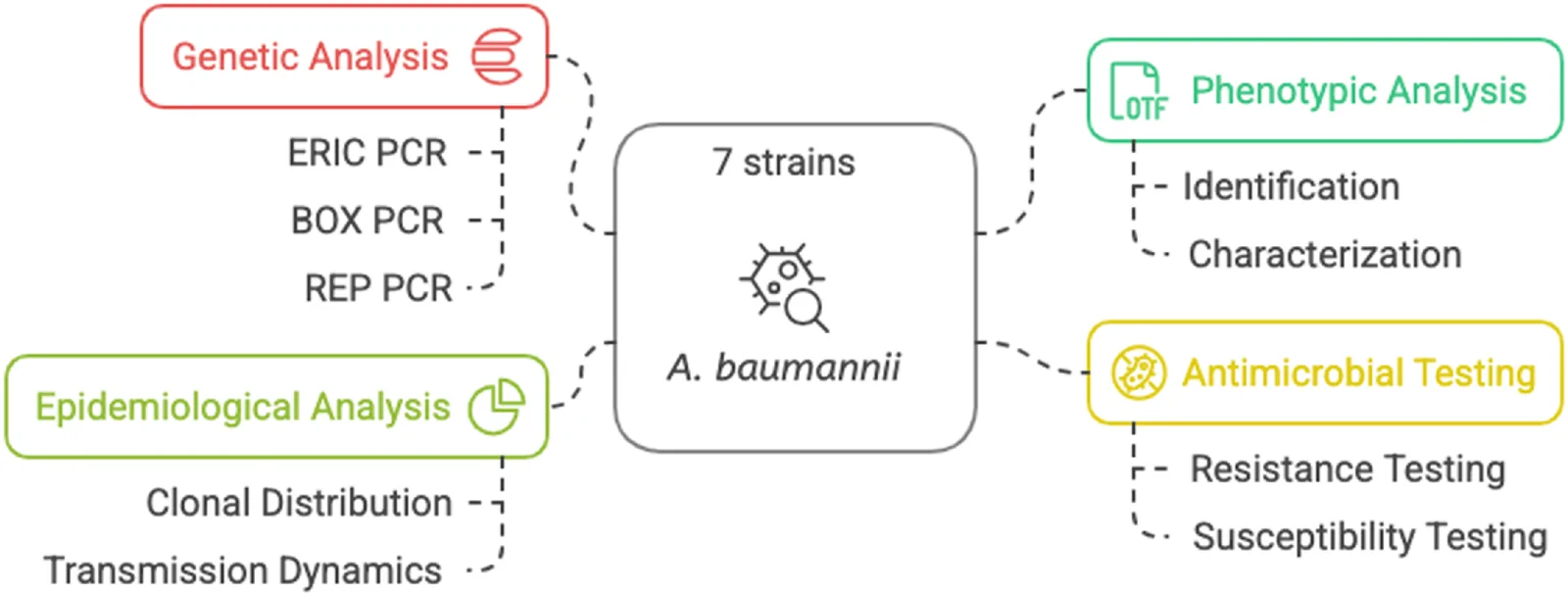
Flowchart of isolate selection and the analytical workflow used in this study.

This retrospective study included seven MDR *A. baumannii* isolates recovered from patients admitted to the ICU of Lambayeque Regional Hospital between April 2015 and September 2021 (Table S1). Eligible isolates were recovered from clinical specimens associated with hospital-acquired infections, defined as infections developing at least 72 h after hospital admission. The available specimens included urine, pus, bronchial fluid, and a vaginal swab. Clinical data were obtained from electronic medical records. Initial identification was based on Gram staining, culture, and biochemical testing with API 20E or the VITEK 2 system (bioMérieux, Durham, NC, USA). Species identification was confirmed by MALDI-TOF MS. Given the exploratory design and small sample size, analyses were primarily descriptive. Because of the limited availability of stored samples during the study period, only multidrug-resistant isolates belonging to the *Acinetobacter baumannii* complex were included. *A. baumannii* is recognized as a critical-priority bacterial pathogen of public health importance [27].

### 2.2 Strain isolation and antimicrobial susceptibility testing

Microorganisms were initially identified with the VITEK 2 Compact system (bioMérieux, Durham, NC, USA). Antimicrobial susceptibility was evaluated by disk diffusion, and inhibition-zone diameters were interpreted according to the Clinical and Laboratory Standards Institute guidelines (CLSI, 2023) [28] (Table S2). Overnight cultures were grown on blood agar at 37 °C. Colonies were suspended in 5 mL of sterile saline (0.85% NaCl) to a 0.5 McFarland standard (approximately 1-2 × 10^8 CFU/mL) and spread evenly on Mueller-Hinton agar. Antimicrobial disks were placed on the agar surface, and plates were incubated at 37 °C for 16-20 h. The tested agents were piperacillin-tazobactam (36 μg, Liofilchem), meropenem (10 μg, Liofilchem), imipenem (10 μg, Liofilchem), gentamicin (10 μg, Oxoid), tobramycin (10 μg, Liofilchem), amikacin (30 μg, Oxoid), cefepime (30 μg, Liofilchem), ceftazidime (10 μg, Liofilchem), levofloxacin (5 μg, Liofilchem), ciprofloxacin (5 μg, Liofilchem), and trimethoprim-sulfamethoxazole (25 μg, Liofilchem). *Pseudomonas aeruginosa* ATCC 27853 and *Escherichia coli* ATCC 25922 were used for quality control. Isolates were stored at −80 °C until molecular analysis.

### *2.3* Repetitive-element PCR typing

Genomic DNA was extracted from each isolate by a phenol-chloroform protocol, quantified at 260 nm with a NanoDrop spectrophotometer (NanoDrop Technologies, Wilmington, DE, USA), and adjusted to approximately 50 ng/μL. REP-PCR used primers REP1R-I (5′-IIIICGICGICATCIGGC-3′) and REP2-I (5′-ICGICTTATCIGGCCTAC-3′) [29]. ERIC-PCR used primers ERIC1R (5′-ATGTAAGCTCCTGGGGATTCAC-3′) and ERIC2 (5′-AAGTAAGTGACTGGGGTGAGCG-3′), and BOX-PCR used primer BOXA1R (5′-CTACGGCAAGGCGACGCTGACG-3′)

Each PCR was performed in a 25 μL reaction containing 1× PCR buffer, 2.5 mM MgCl2, 200 μM of each dNTP, 0.5 μM of each primer, 1.5 U of Taq DNA polymerase, and 50 ng of template DNA. Cycling consisted of initial denaturation at 95 °C for 5 min; 35 cycles of denaturation at 94 °C for 30 s, annealing at 50-55 °C for 1 min according to the primer set, and extension at 72 °C for 1.5 min; and a final extension at 72 °C for 10 min.

PCR products were separated by electrophoresis on 1.5% agarose gels stained with SYBR Safe. A 1 kb DNA ladder was used for size estimation. Bands were scored as present or absent for each BOX-PCR, ERIC-PCR, and REP-PCR profile. Similarity matrices and dendrograms were generated as described in Section 2.5 [30].

### *2.4* MALDI-TOF MS identification and profiling

Bacterial identification was performed by MALDI-TOF MS using the manufacturer's direct-smear extraction protocol and previously described methods [31] Colonies were grown on sheep blood agar (Plastlabor, Brazil) at 37 °C for 24 h. A single colony was transferred to a polished-steel MSP 96 target plate (Bruker Daltonics, Germany), treated with 70% formic acid, and overlaid with a saturated alpha-cyano-4-hydroxycinnamic acid matrix solution (Bruker Daltonics). Mass spectra were acquired in positive linear mode on a Microflex LT instrument (Bruker Daltonics) with ion-source voltages of 2.0 and 1.8 kV, a lens voltage of 6.0 kV, a laser frequency of 60 Hz, and a mass range of 2000–20,000 Da. Spectra were compared with the Bruker Biotyper database (version 3.1); log scores ≥2.0 were considered reliable for species-level identification. The instrument was calibrated with the Bruker Bacterial Test Standard. Main spectral profiles (MSPs) were compared using the MALDI Biotyper dendrogram tool. Dendrogram distances were normalized to a maximum value of 1,000, and spectral correlations were displayed as a heatmap in which warmer colors indicate greater similarity and cooler colors indicate lower similarity [32].

### *2.5* Statistical and similarity analyses

Because this was an exploratory study of seven isolates, results were summarized descriptively. Fisher's exact test was used when applicable, with P < 0.05 considered statistically significant.

For repetitive-element PCR, binary band-presence matrices were analyzed in PAST version 5.0.1 using the Dice similarity coefficient and unweighted pair-group method with arithmetic mean (UPGMA) clustering [30], [33]. Isolates with a similarity coefficient ≥0.85 were assigned to the same genotype. MSP relationships were assessed with the composite correlation index (CCI), which ranges from 0 (low spectral similarity) to 1 (high spectral similarity).

## 3 Results

### 3.1 Antimicrobial susceptibility profiles

All seven *A. baumannii* isolates were resistant to piperacillin-tazobactam, ceftazidime, imipenem, meropenem, ciprofloxacin, and trimethoprim-sulfamethoxazole (Fig. 3; Table S3). Ac-1062 showed intermediate susceptibility to cefepime, whereas the other six isolates were resistant. Responses to aminoglycosides varied: several isolates were susceptible to amikacin or gentamicin, whereas tobramycin results ranged from intermediate to resistant. Levofloxacin susceptibility also varied; Ac-1062 was susceptible, Ac-891 was intermediate, and the remaining isolates were resistant.

**Fig. 3:**
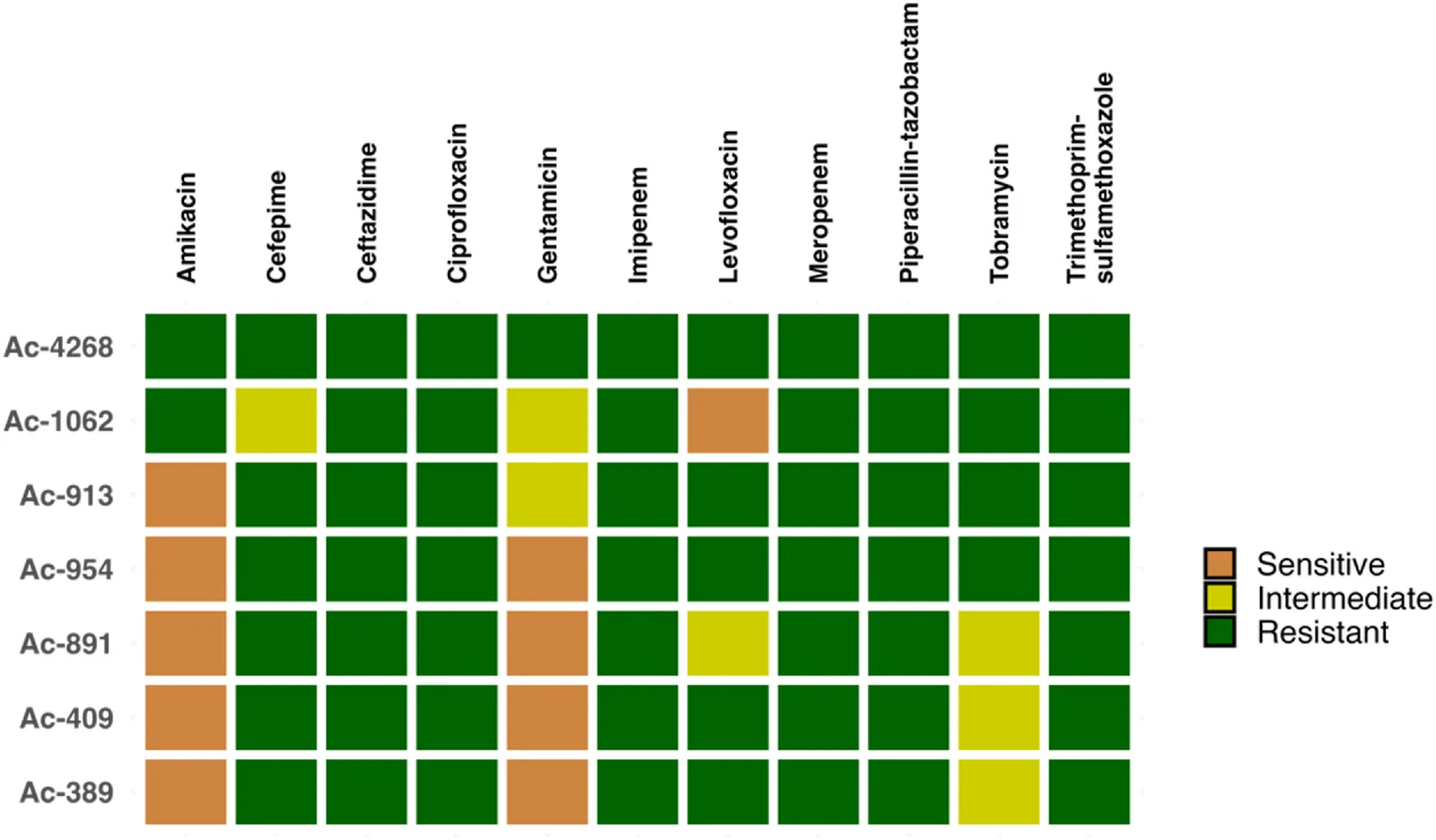
Antimicrobial susceptibility profiles of seven *A. baumannii* isolates. Orange indicates susceptible, yellow indicates intermediate, and green indicates resistant. The tested agents include aminoglycosides, beta-lactams, fluoroquinolones, and a folate-pathway inhibitor.

All isolates met the criteria for MDR. Ac-4268 was resistant to every antimicrobial tested and was classified as XDR. Unlike Ac-4268, the six isolates in the principal repetitive-element PCR group retained susceptibility to at least one tested agent (Fig. 3; Figs. S1–S7). Isolate Ac-4268, which does not belong to the main cluster, showed a markedly different resistance profile, and was resistant to all antibiotics tested (Fig. 3; Figs. S1–S7).

### 3.2 BOX-PCR, ERIC-PCR, and REP-PCR cluster analysis

Seven *A. baumannii* isolates were compared using BOX-PCR, ERIC-PCR, and REP-PCR banding profiles (Fig. 4). UPGMA dendrograms based on Dice similarity identified one group of six isolates (Ac-913, Ac-954, Ac-891, Ac-409, Ac-389, and Ac-1062) at the 0.85 cutoff. Ac-4268 formed a separate profile in all three analyses.

**Fig. 4:**
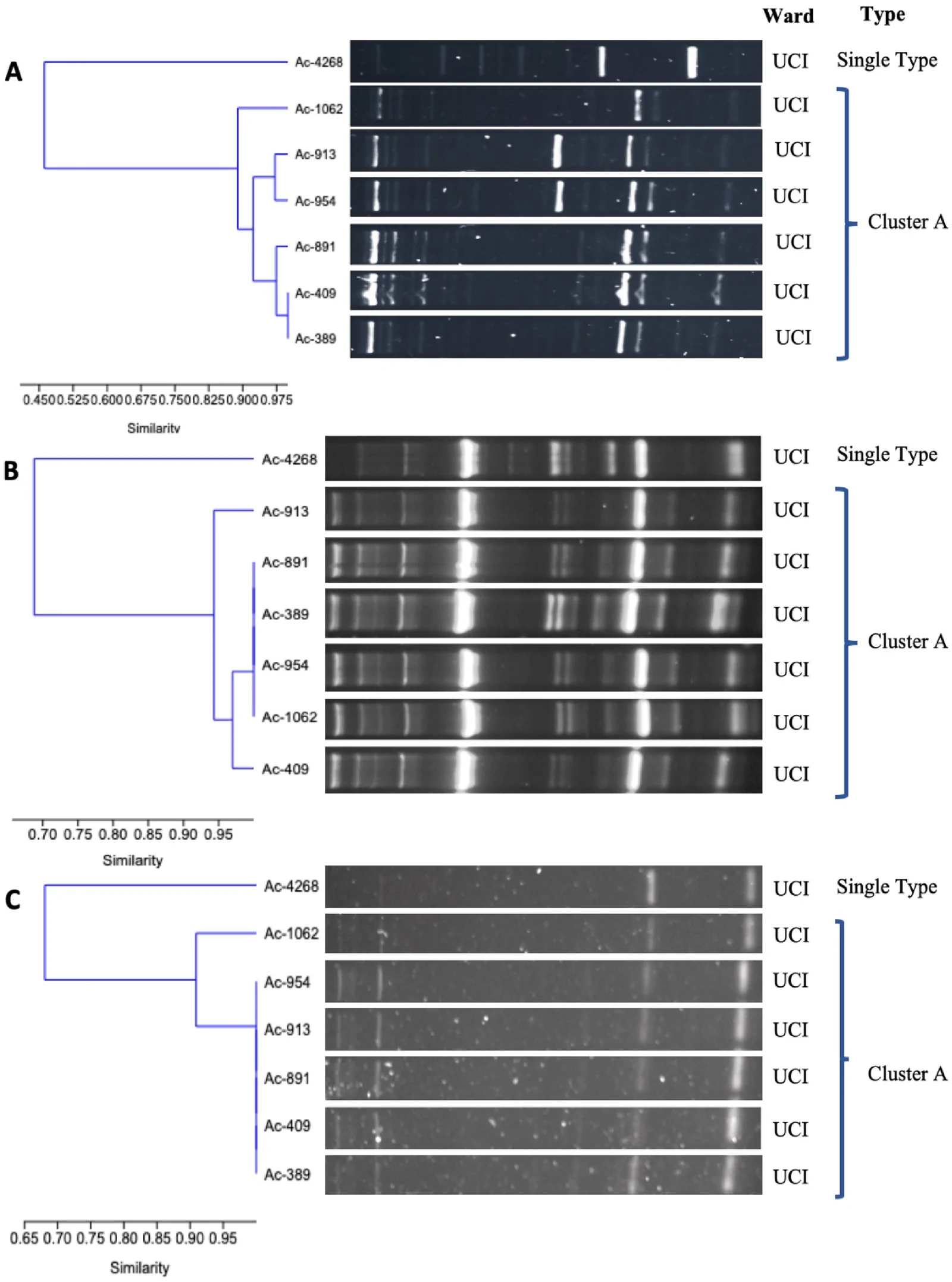
Dendrograms showing genetic similarity among *A. baumannii* isolates based on (A) BOX-PCR, (B) ERIC-PCR, and (C) REP-PCR banding profiles. The similarity scales show the grouping of related profiles; a coefficient of 0.85 was used as the genotype cutoff.

### 3.3 MALDI-TOF MS profiles of antimicrobial-resistant isolates

MALDI-TOF MS identified all seven isolates as *A. baumannii* with log scores >2.0 (Figs. S8–S14). The MSP dendrogram generated in MALDI Biotyper version 3.1 contained one five-isolate cluster (Ac-389, Ac-1062, Ac-913, Ac-409, and Ac-891) and two individual profiles (Ac-954 and Ac-4268) at an arbitrary distance cutoff of 200 (Fig. 5) Ac-954 and Ac-4268 had the greatest distances from the five-isolate cluster.

**Fig. 5:**
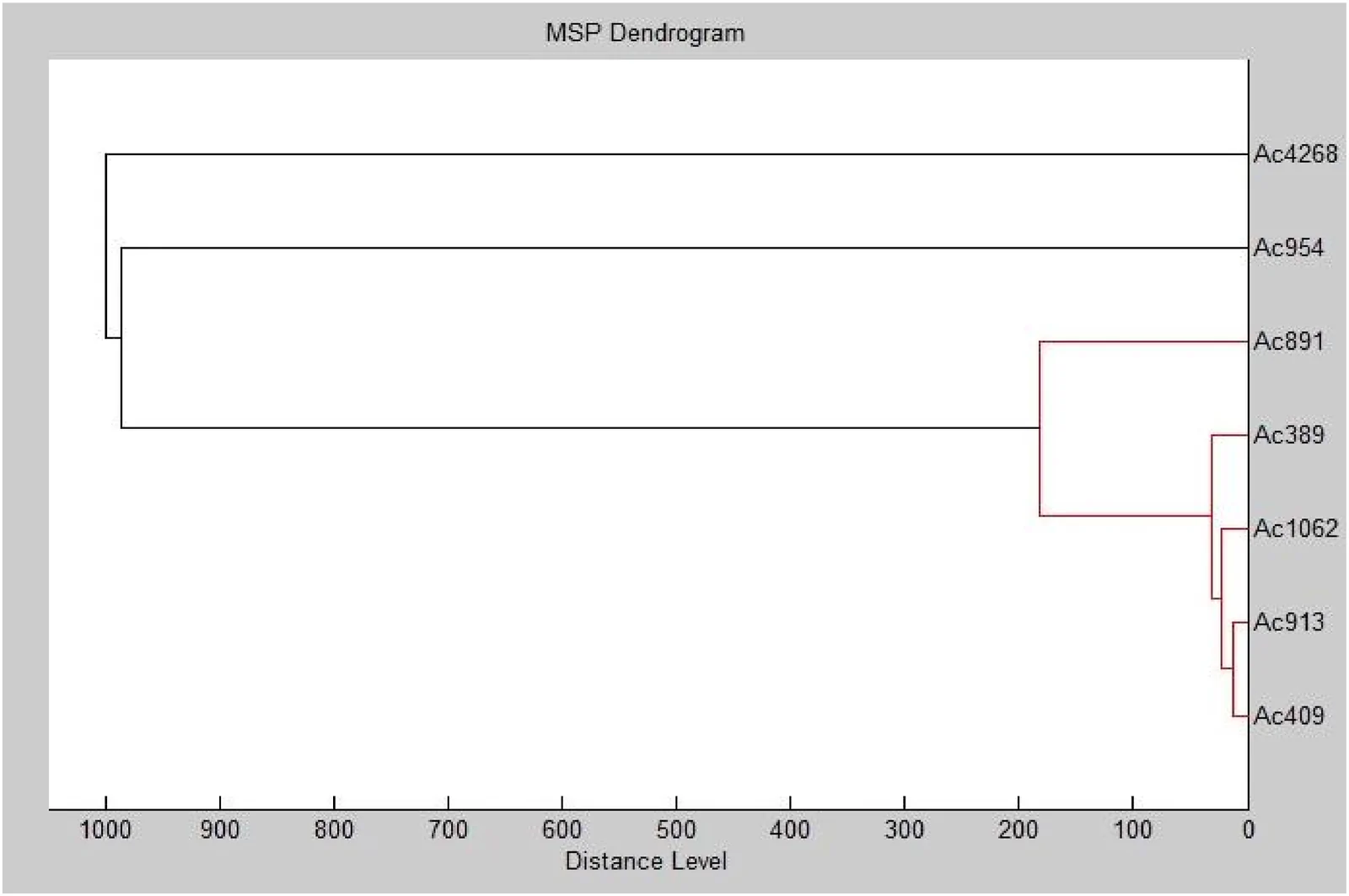
Main spectral profile dendrogram showing one five-isolate cluster and two individual profiles, Ac-954 and Ac-4268. The distance scale ranges from 0 to 1000.

The MSP grouping differed from the repetitive-element PCR grouping because Ac-954 clustered with the six-isolate group by PCR but appeared as an individual profile in the MSP dendrogram.

The CCI heatmap summarizes pairwise spectral similarity among the seven *A. baumannii* isolates (Fig. 6).

**Fig. 6:**
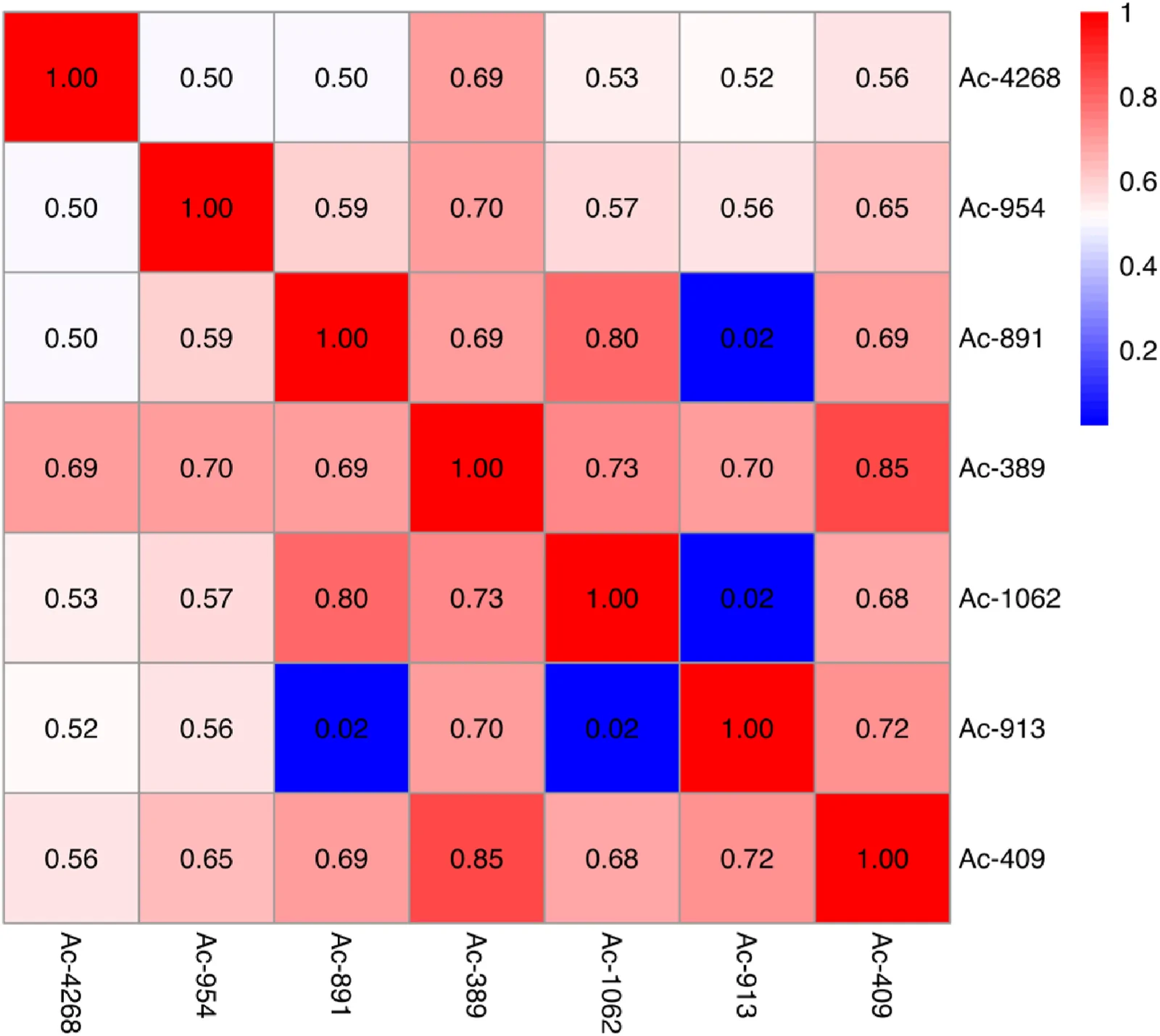
MSP-based heatmap of *A. baumannii* isolates showing spectral similarity from low (blue) to high (red), generated with MALDI Biotyper version 3.1.

Ac-389 and Ac-409 had the highest pairwise MSP similarity (CCI = 0.85), followed by Ac-891 and Ac-1062 (CCI = 0.80). Ac-913 had the lowest values with Ac-891 and Ac-1062 (CCI = 0.02). CCI values between Ac-4268 and the other isolates were approximately 0.50. Fig. 7 shows the seven individual mass spectra.

**Fig. 7:**
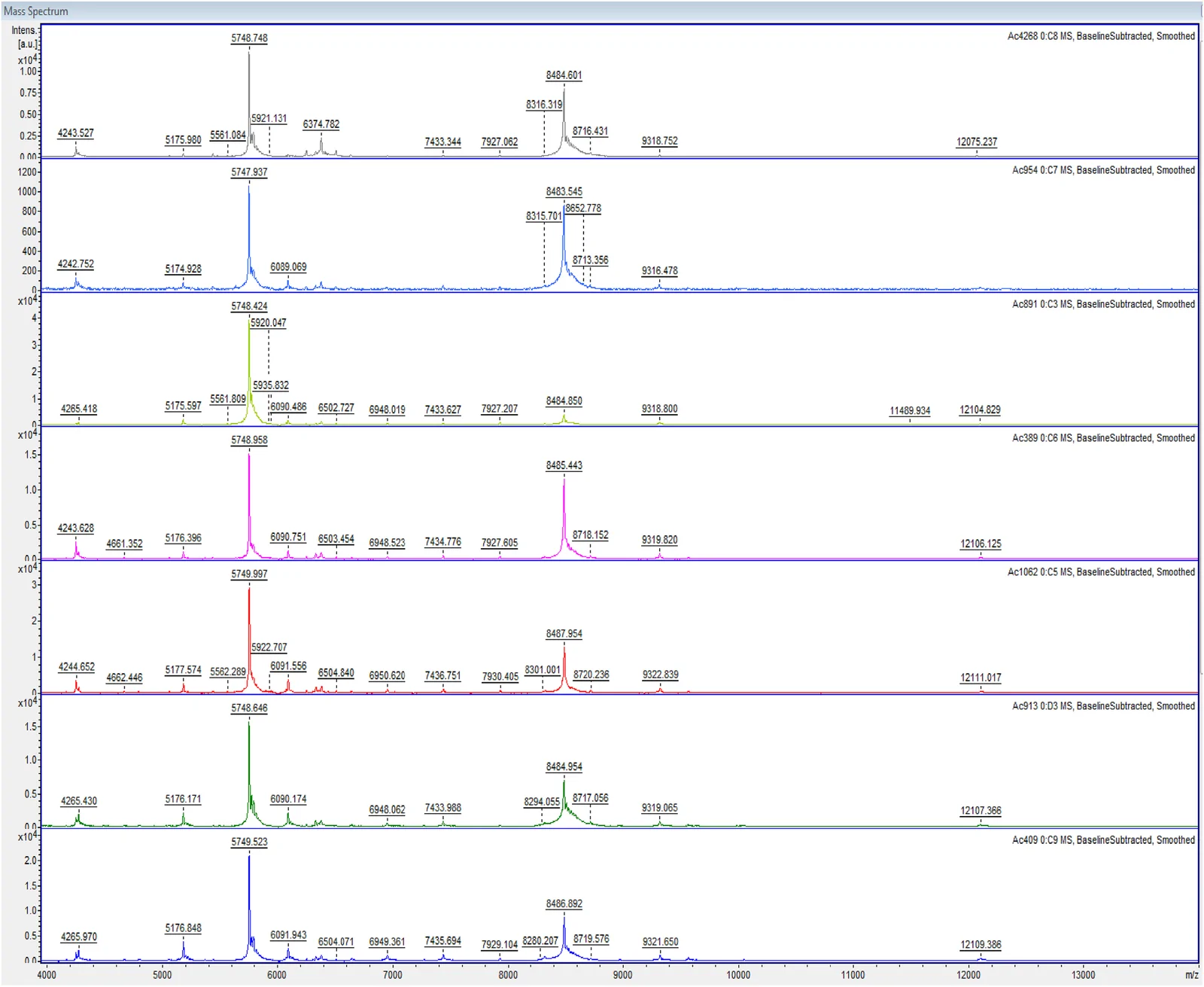
Protein mass spectra of the *A. baumannii* isolates, from top to bottom: Ac-4268, Ac-954, Ac-891, Ac-389, Ac-1062, Ac-913, and Ac-409. Spectra were visualized with flexAnalysis version 3.4 (Bruker Daltonics, Bremen, Germany).

Each spectrum contained characteristic protein or peptide peaks across the measured mass-to-charge (*m*/*z*) range. Peak positions and intensities varied among isolates and formed the basis of the MSP dendrogram and CCI comparisons.

Ac-4268 showed a spectral profile distinct from those of Ac-891 and Ac-954. By comparison, Ac-389, Ac-1062, Ac-913, and Ac-409 showed more similar peak distributions (Fig. 5, Fig. 6).

## 4 Discussion

All seven *A. baumannii* isolates from Lambayeque Regional Hospital were MDR, and Ac-4268 was XDR [34]. This exploratory study was designed to characterize resistance profiles and strain relatedness rather than to provide a comprehensive epidemiological survey. Ac-4268 differed from the other isolates in both repetitive-element PCR and MALDI-TOF MS analyses. It also differed from Ac-1062, which was recovered from the same patient at another anatomical site. These results indicate genomic-fingerprint and proteomic differences but should not be interpreted as a phylogenetic distance. The observed variation is biologically plausible because *A. baumannii* can acquire mutations and mobile genetic elements, including plasmids, transposons, and genomic islands [8], [35]. The six-isolate PCR group is therefore consistent with a predominant related lineage in the hospital, although genomic identity and direct transmission were not established.

The partial agreement between molecular grouping and antimicrobial susceptibility suggests an association between strain relatedness and resistance phenotype, but the small sample does not support a causal inference. Most isolates in the six-isolate PCR group remained susceptible to at least one aminoglycoside, whereas Ac-4268 was resistant to all tested agents. Ac-1062 belonged to the PCR group but showed a distinct susceptibility profile, which could reflect resistance determinants or mutations not resolved by repetitive-element PCR [36], [37], [38], [39]. The five-isolate MSP cluster partly corroborated the PCR results. The close MSP grouping of Ac-1062, Ac-913, and Ac-409 and the high CCI between Ac-389 and Ac-409 may reflect persistence or expansion of a related lineage, but they do not demonstrate direct patient-to-patient transmission.

Regional evidence provides important context for these findings. A multicentre study of nine hospitals in Argentina, Bolivia, Chile, Ecuador, Paraguay, and Uruguay associated most carbapenem-resistant isolates with ST79, ST25, or ST15 and frequently detected *blaOXA-23* [40]. Genomic surveillance in Chile documented the emergence and expansion of ST109, ST162, and ST15/ST318 lineages [13]. In Brazil, OXA-type carbapenemases, particularly OXA-23, have been widely reported in *A. baumannii* [41]. Studies in Colombia described a genetically diverse but clonally structured carbapenem-resistant population [42]. In Peru, international clones II and III were reported among carbapenem-resistant isolates in Lima [43], and a later whole-genome study identified a highly clonal international clone II population in a Peruvian hospital [44]. Genomic analysis in Paraguay identified international clone 2 and international clone 5 isolates, including aminoglycoside-resistance determinants [45]. These studies demonstrate the regional importance of high-risk lineages but do not permit assignment of the Lambayeque isolates to a specific international clone without whole-genome sequencing.

The resistance profiles observed here illustrate the limited therapeutic options for MDR and XDR *A. baumannii*. Recent studies have documented changing epidemiology among patients with *A. baumannii* bacteremia [46]. and high mortality associated with carbapenem-resistant *A. baumannii* bacteremia in ICU cohorts [47]. Prospective multicentre surveillance in Brazil has also documented healthcare-associated bloodstream infections caused by WHO-priority resistant pathogens, including *A. baumannii* [48]. These findings support antimicrobial stewardship and strengthened surveillance in critical-care settings.

The difference between the PCR and MSP classifications of Ac-954 illustrates that the methods measure different biological features. Repetitive-element PCR compares genomic banding patterns, whereas routine MALDI-TOF MS compares abundant protein profiles under defined culture conditions. The two approaches should therefore be interpreted as complementary rather than interchangeable. A study in Taiwan similarly combined MALDI-TOF MS with *blaOXA-51-like* PCR for classification [49]. Specialized MALDI-TOF lipidomic methods can also detect colistin resistance, although that application differs from the MSP analysis used here [50].

Biofilm-associated factors may further contribute to persistence in healthcare environments. The *bap* gene has been studied in MDR, biofilm-producing *A. baumannii* isolates and may be useful in future investigations of virulence and persistence [51] The present study did not measure biofilm formation or *bap* expression, so no inference about their role can be made for these isolates.

Alternative treatment strategies are also under investigation. The IDentif.AI-AMR platform evaluated combinations of approved antimicrobial agents against clinical *A. baumannii* isolates and identified potentially active combinations [52]. Such findings require clinical validation and do not substitute for isolate-specific susceptibility testing or antimicrobial stewardship.

Whole-genome sequencing would provide substantially greater resolution than repetitive-element PCR by identifying sequence types, resistance genes, mutations, and insertion sequences. Large-scale genomic analyses have shown that insertion sequences contribute to antimicrobial resistance in *A. baumannii* [53]. and whole-genome sequencing can identify multiple resistance determinants within clinical isolates [54]. A larger prospective collection would also permit a more reliable assessment of lineage frequency, temporal change, virulence determinants, and epidemiological linkage.

Taken together, the PCR and MSP results support the possible presence of a predominant related lineage and a divergent XDR isolate. The similarity between Ac-389 and Ac-409 and the grouping of several isolates across methods are compatible with localized persistence or expansion, but they are not proof of direct transmission. The divergence of Ac-4268 may reflect the presence of a distinct lineage. These interpretations integrate the molecular and phenotypic findings while recognizing the resolution limits of the methods used.

This study has several limitations. First, only seven of the 24 initially available isolates underwent full molecular and phenotypic characterization. Because selection was guided by preliminary clustering rather than random sampling, the frequency of the predominant lineage may be overestimated or underestimated. The remaining 17 isolates were not preserved for complete downstream analysis and could not be retrospectively characterized. Second, the isolates were collected between 2015 and 2021, so the findings may not represent the current epidemiological situation. Third, whole-genome sequencing was not performed. Repetitive-element PCR and MALDI-TOF MS are accessible methods for preliminary isolate comparison, but they cannot determine multilocus sequence types, resistance-gene content, or transmission chains. Finally, all isolates came from ICU patients at one tertiary referral hospital, and complete data on prior antimicrobial exposure and other risk factors were unavailable. The findings should therefore not be generalized beyond this setting. Prospective sampling, epidemiological linkage data, and whole-genome sequencing are required to confirm whether the inferred lineage persists and to define transmission patterns.

## 5 Conclusion

This study adds molecular epidemiological data on MDR *A. baumannii* from northern Peru. Repetitive-element PCR identified a six-isolate group with similar banding patterns and a divergent isolate, Ac-4268, which also showed an XDR phenotype. MALDI-TOF MS identified one five-isolate MSP cluster and two individual profiles. Together, these findings are consistent with the possible persistence of a predominant related lineage, but they do not confirm clonal transmission.

Repetitive-element PCR and MALDI-TOF MS can support preliminary surveillance in resource-limited settings, although whole genome sequencing is required for definitive lineage assignment and transmission analysis. Because the isolates analyzed here span 2015-2021, a prospective surveillance study incorporating isolates collected after this period is warranted to determine whether the predominant lineage and its resistance profile remain in circulation and to inform current infection control decisions.

## CRediT authorship contribution statement

**Kevin G. Quispe Choque:** Writing – review & editing, Resources. **Nataly Ruiz Quiñones:** Writing – review & editing, Resources. **Milena Binatti Ferreira:** Writing – review & editing, Resources. **Ana L. Perini-Leme-Giordano:** Writing – review & editing, Resources. **Angélica Zaninelli Schreiber:** Writing – review & editing, Resources. **Fabiana Fantinatti Garboggini:** Writing – review & editing, Resources. **Fanny L. Casado:** Writing – review & editing, Resources. **Rene Flores Clavo:** Writing – review & editing, Writing – original draft, Visualization, Supervision, Resources, Project administration, Methodology, Investigation, Funding acquisition, Formal analysis, Conceptualization.

## Ethics approval

The study received the required ethical approval in accordance with applicable institutional and international standards.

## Consent for publication

All authors approved the manuscript and consented to its publication.

## Availability of data and materials

The data supporting the findings of this study are available from the corresponding author upon reasonable request.

## Funding

This research did not receive any specific grant from funding agencies in the public, commercial, or not-for-profit sectors.

## Declaration of competing interest

The authors declare that they have no known competing financial interests or personal relationships that could have appeared to influence the work reported in this paper.

## References

[1] Ibrahim S., Al-Saryi N., Al-Kadmy I.M., Aziz S.N. (2021). Multidrug-resistant Acinetobacter baumannii as an emerging concern in hospitals. Mol Biol Rep.

[2] Garnacho-Montero J., Timsit J.F. (2019). Managing Acinetobacter baumannii infections. Curr Opin Infect Dis.

[3] Liu C., Chen K., Wu Y., Huang L., Fang Y., Lu J., Zhang R. (2022). Epidemiological and genetic characteristics of clinical carbapenem-resistant Acinetobacter baumannii strains collected countrywide from hospital intensive care units (ICUs) in China. Emerg Microb Infect.

[4] Duan Z., Li X., Li S., Zhou H., Hu L., Xia H., Xie F. (2024). Nosocomial surveillance of multidrug-resistant Acinetobacter baumannii: a genomic epidemiological study. Microbiol Spectr.

[5] Roy S., Mukherjee P., Kundu S., Majumder D., Raychaudhuri V., Choudhury L. (2024). Microbial infections in burn patients. Acute Critical Care.

[6] Makke G., Bitar I., Salloum T., Panossian B., Alousi S., Arabaghian H., Tokajian S. (2020). Whole-genome-sequence-based characterization of extensively drug-resistant Acinetobacter baumannii hospital outbreak. mSphere.

[7] Larsson D.J., Flach C.F. (2022). Antibiotic resistance in the environment. Nat Rev Microbiol.

[8] Ntim O.K., Awere-Duodu A., Osman A.H., Donkor E.S. (2025). Antimicrobial resistance of bacterial pathogens isolated from cancer patients: a systematic review and meta-analysis. BMC Infect Dis.

[9] Mulani M.S., Kamble E.E., Kumkar S.N., Tawre M.S., Pardesi K.R. (2019). Emerging strategies to combat ESKAPE pathogens in the era of antimicrobial resistance: a review. Front Microbiol.

[10] Kyriakidis I., Vasileiou E., Pana Z.D., Tragiannidis A. (2021). Acinetobacter baumannii antibiotic resistance mechanisms. Pathogens.

[11] De Souza R.R., Nascimento M.A., Ederli R.B., Santos E.C.M., dos Santos I.A., Ederli J.P.B. (2020). Multi-resistant Acinetobacter baumannii emergence associated with healthcare infections: Meta-analysis. Res Soc Dev.

[12] Hajikhani B., Sameni F., Ghazanfari K., Abdolali B., Yazdanparast A., Dezfuli A.A., Dadashi M. (2023). Prevalence of blaNDM-producing Acinetobacter baumannii strains isolated from clinical samples around the world, a systematic review. Gene Rep.

[13] Brito B.P., Koong J., Wozniak A., Opazo-Capurro A., To J., Garcia P., Hamidian M. (2022). Genomic analysis of carbapenem-resistant Acinetobacter baumannii strains recovered from Chilean hospitals reveals lineages specific to South America and multiple routes for acquisition of antibiotic resistance genes. Microbiol Spectr.

[14] Khoshbakht R., Panahi S., Neshani A., Ghavidel M., Ghazvini K. (2023). Novel approaches to overcome colistin resistance in acinetobacter baumannii: exploring quorum quenching as a potential solution. Microb Pathog.

[15] Bostanghadiri N., Narimisa N., Mirshekar M., Dadgar-Zankbar L., Taki E., Navidifar T., Darban-Sarokhalil D. (2024). Prevalence of colistin resistance in clinical isolates of Acinetobacter baumannii: a systematic review and meta-analysis. Antimicrob Resist Infect Control.

[16] Zhang X., Li F., Awan F., Jiang H., Zeng Z., Lv W. (2021). Molecular epidemiology and clone transmission of carbapenem-resistant Acinetobacter baumannii in ICU rooms. Front Cell Infect Microbiol.

[17] Flores-Paredes W., Luque N., Albornoz R., Rojas N., Espinoza M., Pons M.J., Ruiz J. (2021). Evolution of antimicrobial resistance levels of ESKAPE microorganisms in a Peruvian IV-level hospital. Infect Chemotherapy.

[18] Ramirez M.S., Bonomo R.A., Tolmasky M.E. (2020). Carbapenemases: transforming Acinetobacter baumannii into a yet more dangerous menace. Biomolecules.

[19] Challapa-Mamani M.R., Yareta J., Fajardo-Loyola A., Asmat Marrufo P., Siesquen C.P., Pino-Dueñas J., Marcos-Carbajal P. (2024). Acinetobacter baumannii Co-Resistant to extended spectrum beta-lactamases and carbapenemases in six Peruvian hospital centers. Microbiol Res.

[20] Chilon-Chavez M.A., Muñoz-Inga J.G., Silva-Díaz H. (2022). Perfil microbiológico de microorganismos aislados de pacientes en unidades de cuidados intensivos de un Hospital de Lambayeque, Perú, 2019-2020. Revista de la Facultad de Medicina Humana.

[21] García-Cedrón D., De La Cruz Noriega M., Cabanillas-Chirinos L., Otiniano N.M., Rojas-Villacorta W., Salvatierra-Espinola W., Luján-Velásquez M. (2023). Carbapenemase-producing bacteria isolated from ICU patients of a Peruvian government hospital during the COVID-19 pandemic: a descriptive analysis. Medicina.

[22] Bucka-Kolendo J., Sokołowska B., Winiarczyk S. (2020). Influence of high hydrostatic pressure on the identification of Lactobacillus by MALDI-TOF MS-preliminary study. Microorganisms.

[23] Alnimr A., Alamri A., Alsultan A. (2020). Genetic diversity of imipenem‐resistant Acinetobacter baumannii infections at an intensive care unit. Critical Care Res Practice.

[24] Alamri A.M., Alsultan A.A., Ansari M.A., Alnimr A.M. (2020). Biofilm-formation in clonally unrelated multidrug-resistant Acinetobacter baumannii isolates. Pathogens.

[25] Sepahvand S., Darvishi M., Mokhtari M., Ali Davarpanah M. (2022). Evaluation of genetic diversity of colistin-resistant Acinetobacter baumannii by BOX-PCR and ERIC-PCR: the first report. Future Microbiol.

[26] Pană A.G., Șchiopu P., Țoc D.A., Neculicioiu V.S., Butiuc-Keul A., Farkas A., Junie L.M. (2025). Clonality and the phenotype–genotype correlation of antimicrobial resistance in Acinetobacter baumannii isolates: a multicenter study of clinical isolates from Romania. Microorganisms.

[27] WHO. WHO Bacterial Priority Pathogens List (2024). https://books.google.es/books?id=UjAJEQAAQBAJ&hl=es.

[28] Gaur P., Hada V., Rath R.S., Mohanty A., Singh P., Rukadikar A. (2023). Interpretation of antimicrobial susceptibility testing using European Committee on Antimicrobial Susceptibility Testing (EUCAST) and Clinical and Laboratory Standards Institute (CLSI) breakpoints: analysis of agreement. Cureus.

[29] Versalovic J., Koeuth T., Lupski J.R. (1991). Distribution of repetitive DNA sequences in eubacteria and application to fingerprinting of bacterial genomes. Nucleic Acids Res.

[30] Backeljau T., De Bruyn L., De Wolf H., Jordaens K., Van Dongen S., Winnepenninckx B. (1996). Multiple UPGMA and neighbor-joining trees and the performance of some computer packages. Mol Biol Evol.

[31] Li X., Tang Y., Lu X. (2018). Insight into identification of Acinetobacter species by matrix-assisted laser desorption/ionization time of flight mass spectrometry (MALDI-TOF MS) in the clinical laboratory. J Am Soc Mass Spectrom.

[32] Moussa M., Cauvin E., Le Piouffle A., Lucas O., Bidault A., Paillard C., Garcia C. (2021). A MALDI-TOF MS database for fast identification of Vibrio spp. potentially pathogenic to marine mollusks. Appl Microbiol Biotechnol.

[33] Heras J., Domínguez C., Mata E. (2015). GelJ – a tool for analyzing DNA fingerprint gel images. BMC Bioinf.

[34] Ndiaye I., Sow O., Cissé A., Ba B.S., Thiam F., Boye M.M., Seck A. (2026). Whole genome analysis of Carbapenem Resistant Acinetobacter baumannii isolates from healthcare-associated infections in Dakar, Senegal. New Microbes New Infect.

[35] Ahuatzin-Flores O.E., Torres E., Chávez-Bravo E. (2024). Acinetobacter baumannii, a multidrug-resistant opportunistic pathogen in new habitats: a systematic review. Microorganisms.

[36] Cerezales M., Xanthopoulou K., Wille J., Krut O., Seifert H., Gallego L., Higgins P.G. (2020). Mobile genetic elements harboring antibiotic resistance determinants in Acinetobacter baumannii isolates from Bolivia. Front Microbiol.

[37] Noel H.R., Petrey J.R., Palmer L.D. (2022). Mobile genetic elements in Acinetobacter antibiotic‐resistance acquisition and dissemination. Ann N Y Acad Sci.

[38] Scoffone V.C., Trespidi G., Barbieri G., Arshad A., Israyilova A., Buroni S. (2025). The evolution of antimicrobial resistance in Acinetobacter baumannii and new strategies to fight it. Antibiotics.

[39] Bengtsson-Palme J., Kristiansson E., Larsson D.J. (2018). Environmental factors influencing the development and spread of antibiotic resistance. FEMS Microbiol Rev.

[40] Rodríguez C.H., Balderrama Yarhui N., Nastro M., Nuñez Quezada T., Castro Cañarte G., Magne Ventura R., Famiglietti A. (2016). Molecular epidemiology of carbapenem-resistant Acinetobacter baumannii in South America. J Med Microbiol.

[41] Opazo A., Domínguez M., Bello H., Amyes S.G., González-Rocha G. (2012). OXA-type carbapenemases in *Acinetobacter baumannii* in South America. J Infect Dev Ctries.

[42] Correa A., Del Campo R., Escandón-Vargas K., Perenguez M., Rodríguez-Baños M., Hernández-Gómez C., Villegas M.V. (2018). Distinct genetic diversity of carbapenem-resistant *Acinetobacter baumannii* from Colombian hospitals. Microb Drug Resist.

[43] Levy-Blitchtein S., Roca I., Plasencia-Rebata S., Vicente-Taboada W., Velásquez-Pomar J., Muñoz L., Vila J. (2018). Emergence and spread of carbapenem-resistant *Acinetobacter baumannii* international clones II and III in Lima, Peru. Emerg Microb Infect.

[44] Pons M.J., Guibert F., Choque-Matos J., Palomino-Kobayashi L.A., Fernandez-Castro P., Gonzales P., Ruiz J. (2025). Highly clonal carbapenem-resistant *Acinetobacter baumannii* isolates from a Peruvian hospital. Microbiol Spectr.

[45] Sánchez-Urtaza S., Alfonso-Alarcón L., Arazo del Pino R., Burgwinkel T., Ocampo-Sosa A., Gonzalez R., Gallego L. (2025). Genomic characterization of multidrug-resistant clinical *Acinetobacter baumannii* isolates from a hospital in Paraguay. Front Cell Infect Microbiol.

[46] Chotiprasitsakul D., Ao-udomsuk K., Santinirand P. (2025). Epidemiology and microbiological trend of Acinetobacter baumannii bacteremia. Int J Infect Dis.

[47] Karakonstantis S., Kritsotakis E.I., Tziolos R.N., Vassilopoulou L., Loukaki M., Kypraiou D., Kofteridis D.P. (2025). Mortality due to carbapenem-resistant Acinetobacter baumannii bacteraemia: a five-year cohort study in intensive care patients. Clin Microbiol Infection.

[48] Antochevis L.C., Wilhelm C.M., Arns B., Sganzerla D., Sudbrack L.O., Nogueira T.C., de Oliveira J.S. (2025). World Health Organization priority antimicrobial resistance in Enterobacterales, Acinetobacter baumannii, Pseudomonas aeruginosa, Staphylococcus aureus and Enterococcus faecium healthcare-associated bloodstream infections in Brazil (ASCENSION): a prospective, multicentre, observational study. Lancet Reg Health, Am.

[49] Quyen T.L.T., Hsieh Y.C., Li S.W., Wu L.T., Liu Y.Z., Pan Y.J. (2025). Molecular epidemiology of carbapenem-resistant Acinetobacter baumannii group in Taiwan. mSphere.

[50] Dortet L., Potron A., Bonnin R.A., Plesiat P., Naas T., Filloux A., Larrouy-Maumus G. (2018). Rapid detection of colistin resistance in Acinetobacter baumannii using MALDI-TOF-based lipidomics on intact bacteria. Sci Rep.

[51] Kakavan M., Gholami M., Ahanjan M., Ebrahimzadeh M.A., Salehian M., Roozbahani F., Goli H.R. (2025). Expression of bap gene in multidrug-resistant and biofilm-producing Acinetobacter baumannii clinical isolates. BMC Microbiol.

[52] You K., Binte Mohamed Yazid N., Chong L.M., Hooi L., Wang P., Zhuang I., Ho D. (2025). Flash optimization of drug combinations for Acinetobacter baumannii with IDentif. AI-AMR npj Antimicrobial Resistance.

[53] Xie F., Wang L., Li S., Hu L., Wen Y., Li X., Xie L. (2025). Large-scale genomic analysis reveals significant role of insertion sequences in antimicrobial resistance of Acinetobacter baumannii. mBio.

[54] Habibi N., Mustafa A.S., Nasser K., Al-Obaid I., Alfouzan W., Uddin S., Khan M.W. (2025). Genomic characterization and identification of multiple drug resistance genes in clinical isolates of Acinetobacter baumannii through whole genome sequencing. Mol Biol Rep.

