# Supplementary material for: Molecular evidence of a dominant multidrug resistant Acinetobacter baumannii lineage in a tertiary hospital in northern Peru
Source: New Microbes New Infect. 2026 Oct 10;74:101864. doi: 10.1016/j.nmni.2026.101864 (PMC13694419; doi:10.1016/j.nmni.2026.101864)
Supplement: Supplementary file 1 [file mmc1.docx]

**Supplementary materials**

The supplementary tables and figures associated with this article are provided below

**Table S1.** *A. baumannii* isolate codes, clinical diagnoses, ICU procedures, and sampling dates.

| A. Isolate code | Clinical diagnosis | ICU procedure | Sampling date |
| --- | --- | --- | --- |
| Ac-913 | Moderate-to-severe traumatic brain injury and hospital-acquired pneumonia. | Mechanical ventilation | 13/07/2015 |
| Ac-954 | Ischemic stroke, hospital-acquired pneumonia, and sepsis. | Mechanical ventilation and nasogastric tube placement | 17/07/2017 |
| Ac-891 | Hepatic septic shock and hospital-acquired infection. | Hepatic abscess drainage | 09/07/2018 |
| Ac-409 | Severe traumatic brain injury and multiple-organ failure. | Mechanical ventilation | 18/09/2019 |
| Ac-389 | Ovarian adenocarcinoma and hospital-acquired infection. | Mechanical ventilation | 20/02/2020 |
| Ac-1062 | Sepsis of abdominal origin. | Exploratory laparotomy | 09/09/2021 |
| Ac-4268 | Sepsis due to intestinal obstruction. | Exploratory laparotomy | 21/09/2021 |

**Table S2**. Interpretive categories and disk-diffusion zone-diameter breakpoints (mm) according to CLSI guidelines.

| Class | Antimicrobial agent | S | I | R |
| --- | --- | --- | --- | --- |
| BETA-LACTAM COMBINATION AGENTS | Piperacillin-tazobactam | ≥ 21 | 18–20 | ≤17 |
| CEPHALOSPORINS | Ceftazidime | ≥18 | 15-17 | ≤14 |
|  | Cefepime | ≥18 | 15-17 | ≤14 |
| CARBAPENEMS | Imipenem | ≥18 | 15-17 | ≤14 |
|  | Meropenem | ≥18 | 15-17 | ≤14 |
| AMINOGLYCOSIDES | Gentamicin | ≥15 | 13-14 | ≤12 |
|  | Tobramycin | ≥15 | 13-14 | ≤12 |
|  | Amikacin | ≥17 | 15-16 | ≤14 |
| FLUOROQUINOLONES | Ciprofloxacin | ≥21 | 16-20 | ≤15 |
|  | Levofloxacin | ≥17 | 14-16 | ≤13 |
| FOLATE PATHWAY ANTAGONISTS | Trimethoprim- sulfamethoxazole | ≥16 | 11-15 | ≤10 |

**Table S3**. Antimicrobial susceptibility results for seven *A. baumannii* isolates interpreted according to CLSI criteria.

|  | Antimicrobial agents | | | | | | | | | | |  |
| --- | --- | --- | --- | --- | --- | --- | --- | --- | --- | --- | --- | --- |
| Strain | **Piperacillin-tazobactam** | **Ceftazidime** | **Cefepime** | **Imipenem** | **Meropenem** | **Gentamicin** | **Tobramycin** | **Amikacin** | **Ciprofloxacin** | **Levofloxacin** | **Trimethoprim- sulfamethoxazole** | |
| Ac-389 | R | R | R | R | R | S | I | S | R | R | R |  |
| Ac-409 | R | R | R | R | R | S | I | S | R | R | R |  |
| Ac-891 | R | R | R | R | R | S | I | S | R | I | R |  |
| Ac-913 | R | R | R | R | R | I | R | S | R | R | R |  |
| Ac-954 | R | R | R | R | R | S | R | S | R | R | R |  |
| Ac-1062 | R | R | I | R | R | I | R | R | R | S | R |  |
| Ac-4268 | R | R | R | R | R | R | R | R | R | R | R |  |
| R, resistant; I, intermediate; S, susceptible | | | | | | | | | | | |  |

**Figure S1.** Disk-diffusion susceptibility test for *A. baumannii* isolate Ac-913.

**Figure S2.** Disk-diffusion susceptibility test for *A. baumannii* isolate Ac-954.

**Figure S3.** Disk-diffusion susceptibility test for *A. baumannii* isolate Ac-891.

**Figure S4.** Disk-diffusion susceptibility test for *A. baumannii* isolate Ac-409.

**Figure S5.** Disk-diffusion susceptibility test for *A. baumannii* isolate Ac-389.

**Figure S6.** Disk-diffusion susceptibility test for *A. baumannii* isolate Ac-1062.

**Figure S7.** Disk-diffusion susceptibility test for *A. baumannii* isolate Ac-4268.

**Figure S8.** Taxonomic identification data for *A. baumannii* isolate Ac-891, including the identification number and NCBI accession code.

**Figure S9.** Taxonomic identification data for *A. baumannii* isolate Ac-1062, including the identification number and NCBI accession code.

**Figure S10.** Taxonomic identification data for *A. baumannii* isolate Ac-389, including the identification number and NCBI accession code.

**Figure S11.** Taxonomic identification data for *A. baumannii* isolate Ac-954, including the identification number and NCBI accession code.

**Figure S12.** Taxonomic identification data for *A. baumannii* isolate Ac-4268, including the identification number and NCBI accession code.

**Figure S13.** Taxonomic identification data for *A. baumannii* isolate Ac-409, including the identification number and NCBI accession code.

**Figure S14.** Taxonomic identification data for *A. baumannii* isolate Ac-913, including the identification number and NCBI accession code.
